# Book Reviews

**Published:** 1897-11

**Authors:** 


### Book Reviews.

A System of Practical Medicine. By American authors. Edited by Alfred Lee Loomis, M. D., Late Professor of Pathology and Practical Medicine in the New York University, and William Gilman Thompson, M. D., Professor of Materia Medica, Therapeutics and Clinical Medicine in the New York University. Volume II. Philadelphia and New York: Lea Brothers & Co., Publishers.

This second volume of an American system of practical medicine (the first of which was noticed in the May edition of this journal) deals with diseases of the respiratory and circulatory systems, of the mediastinum, of the blood, of the kidneys, of the bladder and of the prostate gland. The consideration of diseases of the respiratory system occupies the first 325 pages of the book and the six contributors are S. Edwin Solly, Elbridge G. Cutler, Reginald H. Fitz, A. Lawrence Mason, William Whitworth Gannett and Herbert B. Whitney. The respiratory tract has rarely been so exhaustively considered with reference to its diseases as in this section and never more ably.

The next section, pages 327 to 633, is devoted to the consideration of diseases of the circulatory system and of the mediastinum. Elbridge G. Cutler writes of physical signs of cardiac disease, cardiac atrophy, cardiac rupture, acute and chronic myocarditis and the fat heart; Warren Coleman, of pericarditis and hypertrophy and dilatation of the heart; Alfred Lee Loomis, of endocarditis; Charles E. Quimby, of cardiac thrombosis and aneurism, morbid growths and parasites, hydropericardium, pneumopericardium, syphilis of the heart, wounds of the heart and foreign bodies of the heart; James T. Whittaker, of neuroses of the heart and of diseases of the blood-vessels; and finally, Irving S. Haynes discourses upon diseases of the mediastinum.

It is stated that the chapter written by Dr. Loomis on endocarditis is one of his last literary efforts. The subject is forcefully presented in the author's usual terse and graphic style and is one of the best expositions of the subject yet offered to the profession.

Diseases of the blood occupy the next section, which is contributed by Frederick C. Shattuck and Richard C. Cabot. The titles are: examination of the blood, plethora, anemia, leucemia, leucocytosis, Hodgkin's disease, and the blood in infancy.

In the section devoted to the consideration of diseases of the kidneys, Henry P. Loomis writes on nephritis, amyloid degeneration of the kidneys, and renal hyperemia; I. N. Danforth discourses upon pyelitis, renal calculus, hydronephrosis, renal tumors, renal abscess, perirenal abscess, renal parasites and neuroses of the kidney, while James Tyson tells us of abnormalities of form and position of the kidney and of renal inadequacy.

The last section treats of diseases of the bladder and prostate gland. In this I. N. Danforth again appears as an author, this time discoursing upon diseases of the bladder and prostatitis, while Thomas D. Coleman closes the volume with chapters on abnormalities of the urine and uremia.

From all this array of authors and titles it will be observed that the editors have been surrounded by a group of leaders in medicine, who have yielded them loyal aid in completing this great volume. It is illustrated on a fitting scale with numerous engravings and plates that amplify the text and are specimens of artistic worth. The literature of medicine has been greatly enriched by this volume.

A Text-Book of Diseases of Women. By Charles B. Penrose, M. D., Ph. D., Professor of Gynecology in the University of Pennsylvania; Surgeon to the Gynecean Hospital, Philadelphia. Octavo, pp. 529. Philadelphia: W. B. Saunders, 925 Walnut street. 1897.

This book exhibits the personal opinions of the author based on an experience which has been considerable. It is intended for the medical student and the general practitioner of medicine who has little or no experience in gynecological work. The author has in most instances recommended but one plan of treatment for each disease; in this way he avoids confusing the audience the book is intended to reach. Only such facts of anatomy, physiology and pathology not found in the general text-books are included here, and then only when it appears important for the clear understanding of the subject dealt with.

There may be, doubtless are, those who will differ from the author on some or a number of the subjects considered in this volume, but none will question his clearness of understanding or expression. This makes the book a valuable one to consult, and when contrasted with others that leave one in doubt as to what procedure to adopt at a particular juncture, it must easily take rank with the best of modern text-books on gynecology.

There is no drouth at present of works on gynecology, and he must be a courageous man who would put forth another at this time; but when a man has something to say, then says it, and stops when he has finished, he will always be sure of attentive listeners. This is just what Penrose has done and his attentive audience must necessarily be a large one. We do not expect to enter into an analysis at this time of this work, but will embrace the opportunity to speak of two or three excellent points which impress us with especial force.

In describing the methods of examination the author is particularly clear and concise and has given appropriate illustrations in the various stages of procedure. Again, we have rarely seen injuries of the perineum and displacements of the womb as well handled as in this book. Here, too, the illustrations are most appropriate and amplify the text in the clearest possible manner. Even the novitiate, after reading these chapters, cannot fail to have a reasonably clear comprehension of the subject. Lacerations of the cervix are always difficult for the student to comprehend, especially as regards their repair, but they are here set forth in such a manner as to bring them within the comprehension of the dullest mind.

Passing now to the technique of operations upon the uterus and appendages we find a chapter of unusual interest, treated with intelligence and illustrated with great skill. Especially to be commended are the author's description and illustrations of vaginal hysterectomy.

Finally, a word of praise should be spoken of the size and make-up of the book. It is convenient to handle, the paper and type make it pleasing to the eye and the illustrations for the most part are of a high class.

THE MENOPAUSE. A consideration of the phenomena which occur to women at the close of the child-bearing period, with incidental allusions to their relationship to menstruation. Also a particular consideration of the premature (especially the artificial) menopause. By ANDREW F. CURRIER, A. B., M. D., New York City. Duodecimo, pp. xvi.—309. New York: D. Appleton. 1897.

The subject of this book is an interesting one. It is one also that is too little understood or too greatly misunderstood by the great majority of physicians. Dr. Currier is a painstaking writer and he has chosen for his title a subject that he well understands and one in which he has given a vast amount of information. Every general practitioner of medicine should study it with unusual care. It is to such that the majority of women suffering from ills, fancied or supposed, relating to the menopause usually apply. Dr. Currier has conferred lasting benefits upon physicians and patients in presenting his well-timed and scholarly work.

---

INTERNATIONAL CLINICS. A Quarterly of Clinical Lectures on Medicine, Neurology, Surgery, Gynecology, Obstetrics, Ophthalmology, Laryngology, Pharyngology, Rhinology, Otology and Dermatology, and specially prepared articles on treatment. By professors and lecturers in the leading medical colleges of the United States, Germany, Austria, France, Great Britain and Canada. Edited by JUDSON DALAND, M. D., (Univ. of Penna.) Philadelphia, Instructor in Clinical Medicine and Lecturer on Physical Diagnosis in the University of Pennsylvania; Assistant Physician to the Hospital of the University of Pennsylvania, etc.; Fellow of the College of Physicians of Philadelphia. J. Mitchell Bruce, M. D., F. R. C. P., London, England, Physician to, and Lecturer on, the Principles and Practice of Medicine at the Charing Cross Hospital. David W. Finlay, M. D., F. R. C. P., Aberdeen, Scotland, Professor of Practice of Medicine in the University of Aberdeen; Physician to, and Lecturer on, Clinical Medicine in the Aberdeen Royal Infirmary, etc. Volume II. Seventh series. 1897. Octavo, pp. xii.—371. Philadelphia: J. B. Lippincott Co. 1897.

If the value of these clinics is based upon the lecture of G. Ernest Herman on the treatment of puerperal eclampsia they would play a small part in literature of medicine. By quoting Herman's definition of eclampsia our readers will fully comprehend the force and import of the foregoing remark.

Herman says: "By puerperal eclampsia I understand epileptiform convulsions in a pregnant, parturient or puerperal woman whose urine is solid with albumen." The lecture abounds with expressions equally as valueless and quite as misleading as is this one.

But, happily, there are others on which to base a judgment of value. The average of the volume, though somewhat below many of its predecessors, yet contains much that will interest and instruct the reader. The clinics have come so irregularly to us of late that we find the thread somewhat broken and had begun to wonder if they had been discontinued. It, however, appears that they are still alive, as indicated by the one before us.

---

TWENTIETH CENTURY PRACTICE. An International Encyclopedia of Modern Medical Science. By leading authorities of Europe and America. Edited by THOMAS L. STEDMAN, M. D., New York City. In twenty volumes. Volume XI. Diseases of the Nervous System. New York: William Wood & Co. 1897.

This volume is devoted to diseases of the spinal cord, cranial and peripheral nerves, and is ably edited by L. Bruns, of Hanover, Germany, Drs. F. X. Dercum, Lightner Witmer, James Hendrie Lloyd, and Charles K. Mills, of Philadelphia, Paul J. Möbius, F. Winscheid, of Leipsic, and Adolf von Strümpell, of Erlangen.

By far the greater portion of this volume is edited by Dr. Lloyd, who writes on diseases of the cerebro-spinal and sympathetic nerves. The chapters on the trophoneuroses is ably written by Dr. Mills; scleroderma, acromegaly and adiposa dolorosa by Dr. Dercum; diseases of the spinal cord by L. Bruns and F. Winscheid; tabes by P. Möbius, and the combined system diseases of the spinal cord by Adolf Strümpell and the chapter on pain by Lightner Witmer.

The discussion of these subjects is very thorough and scientific and the authors review the literature of the subject to date.

The appearance and typography is similar to the other volumes of this series.

---

THE LIVER OF DYSPEPTICS and Particularly the Cirrhosis Produced by Auto-Intoxication of Gastro-Intestinal Origin. By DR. EMILE BOIX, of Paris. Authorised translation from the latest French edition by PAUL RICHARD BROWN, M. D., Major and Surgeon U. S. Army. New York: G. P. Putnam's Sons. 1897.

With improved knowledge relating to systemic intoxication comes the necessity for increased facilities to differentiate, diagnosticate and eliminate. In other words, we experience in the daily practice of medicine an evergrowing necessity for improvement in our methods of treating gastro-intestinal disorders. The liver is an old offender and has been made the scapegoat for the ignorance of physicians since a very early day.

In this work the dyspeptic liver is handled by a man of science on a scientific basis. There is no reason why every physician should not familiarise himself with the anatomic, pathologic and pathogenic conditions of this organ and learn how to diagnosticate and treat them. This work of Boix will be found an intelligent aid toward the accomplishment of that purpose. It is one of the best monographs that has been issued in regard to the liver and its diseases and no man can expect to master the dyspepsias until he has familiarised himself with the subject dealt with in this volume.

---

Annual of the Universal Medical Sciences and Analytical Index. A yearly report of the progress of the general sanitary sciences throughout the world. Edited by Chas. E. Sajous, M. D., and seventy associate editors, assisted by over 200 corresponding editors, collaborators and correspondents. Illustrated with chromo-lithographs, engravings and maps, in five octavo volumes of about 500 pages each. Philadelphia: F. A. Davis & Co., publishers. 1896.

The new features introduced into this series of the annual are of commendable worth. The length of the abstracts has been increased the better to convey an author's meaning and this has made it necessary to rearrange the entire text. The associate editors have in many instances added personal comments to the articles to which they have attached their initials. Comments bearing no initials were written by the editor. This improvement, in the form of the abstracts, has involved the addition of more than half a million of words.

An analytical index and cyclopedia of treatment, filling about 350 pages, has been added for the benefit of overworked physicians who may not have time to consult more lengthy articles. There are a number of minor improvements which help to increase the general value of the annual.

The Journal has heretofore stated its opinion of the annual, and it only needs here to reaffirm its former judgment with the added statement that the editor and his assistants and associates have year by year widened its scope and improved its material until the series of 1896 has become as near perfect as it is possible to make such a vast work.

---

Reports of Inspections of National, State and Local Quarantine Stations. From annual report Marine Hospital Service. Washington: Government Printing Office. 1897.

This extract of the supervising surgeon-general's annual report contains whatever is of special interest in it pertaining to public health. Sanitary officers will be particularly desirous of obtaining it for the scientific quarantine information it contains. The government at Washington ought to issue bulletins on scientific medical subjects at short intervals. It is hoped that in the near future a Minister of Health will be accorded a seat in the cabinet, with ample power to disseminate information and promulgate orders that will conserve the public health in the best manner known to science. Meanwhile, we must be content, even glad, to receive such bits of official scientific information as such reports as this afford us.

---

THE AMERICAN TEXT-BOOK OF OPERATIVE DENTISTRY. In contributions by eminent American authorities. Edited by EDWARD C. KIRK, D. D. S., Professor of Clinical Dentistry, University of Pennsylvania, Department of Dentistry. Philadelphia and New York: Lea Brothers & Co., Publishers.

The practice of dentistry has improved so much within the past ten years that its literature must necessarily be rewritten because much of it has become obsolete. Dentists, like doctors, must now have passed through preliminary training, fixed collegiate courses, and final examination by the state after graduation, before they can be licensed to practise.

This work is written to supply the necessities of advanced training and education. The editor is an experienced dentist, as well as a competent teacher, and he has surrounded himself with an able group of contributors. The practising dentist will necessarily rely upon this work for much-needed advice in doubtful cases and the student will also find in it just what he requires in his early studies. It is well illustrated and printed on excellent paper with good-faced type. It will easily become adopted as a text-book in all the dental colleges and, necessarily, must be found on the shelves of every progressive dentist.

---

MEDICAL AND SURGICAL REPORT OF THE PRESBYTERIAN HOSPITAL IN THE CITY OF NEW YORK. Volume II. January, 1897. Edited by ANDREW J. MCCOSH, M. D., and WALTER B. JAMES, M. D. New York. 1897.

This report, bound in boards, contains 172 pages and is one of the most elaborate and complete reports issued by any hospital within our knowledge. The book contains nothing but scientific matter and is well edited and printed. There are twenty-six articles and about fifty illustrations; many of the latter are devoted to the rooms and instruments relating to surgical technique. It is a book that may well serve as a model for hospitals. Too little attention is paid to these reports as a rule by members of hospital staffs and they are rarely bound in a form that permits of their preservation in the library.

